# Proteomics of *Brucella*: Technologies and Their Applications for Basic Research and Medical Microbiology

**DOI:** 10.3390/microorganisms8050766

**Published:** 2020-05-20

**Authors:** Gamal Wareth, Mathias W. Pletz, Heinrich Neubauer, Jayaseelan Murugaiyan

**Affiliations:** 1 Friedrich-Loeffler-Institut, Institute of Bacterial Infections and Zoonoses, Naumburger Str. 96a, 07743 Jena, Germany; heinrich.neubauer@fli.de; 2 Institute for Infectious Diseases and Infection Control, Jena University Hospital, Am Klinikum 1, 07747 Jena, Germany; mathias.pletz@med.uni-jena.de; 3 Faculty of Veterinary Medicine, Benha University, Moshtohor, Toukh 13736, Egypt; 4 Institute of Animal Hygiene and Environmental Health, Centre for Infectious Medicine, Freie Universität Berlin, Robert-von-Ostertag-Str. 7-13, 14163 Berlin, Germany; Jayaseelan.murugaiyan@fu-berlin.de; 5 Department of Biotechnology, SRM University AP, Neerukonda, Mangalagiri, Andhra Pradesh 522502, India

**Keywords:** brucellae, proteomics, applications, diagnosis, perspective

## Abstract

Brucellosis is a global zoonosis caused by Gram-negative, facultative intracellular bacteria of the genus *Brucella* (*B.*). Proteomics has been used to investigate a few *B. melitensis* and *B. abortus* strains, but data for other species and biovars are limited. Hence, a comprehensive analysis of proteomes will significantly contribute to understanding the enigmatic biology of brucellae. For direct identification and typing of *Brucella*, matrix-assisted laser desorption ionization—time of flight mass spectrometry (MALDI—TOF MS) has become a reliable tool for routine diagnosis due to its ease of handling, price and sensitivity highlighting the potential of proteome-based techniques. Proteome analysis will also help to overcome the historic but still notorious *Brucella* obstacles of infection medicine, the lack of safe and protective vaccines and sensitive serologic diagnostic tools by identifying the most efficient protein antigens. This perspective summarizes past and recent developments in *Brucella* proteomics with a focus on species identification and serodiagnosis. Future applications of proteomics in these fields are discussed.

## 1. Introduction

Brucellosis is one of the most frequent bacterial zoonoses spread worldwide. The infection is most often acquired via the consumption of unpasteurized milk and dairy products or direct or indirect contact with infected animals or their excretes [1]. The main symptom in animals is abortion, and consequently, the disease was named infectious abortion, contagious abortion, enzootic abortion or Bang’s disease. In humans, the cardinal symptom is fever resulting in names like Crimean fever, Mediterranean fever, rock fever, undulant fever or Malta fever. Finally, the term ‘brucellosis’ was established to clarify the etiology of a manifold syndrome. To date, the genus *Brucella* (*B.*) encompasses 12 accepted nomo-species. The classical six species are *B. abortus*, *B. melitensis*, *B. suis*, *B. canis*, *B. ovis* and *B. neotomae*, which were primarily isolated from bovines, small ruminants, pigs, dogs, sheep and desert woodrats, respectively [2]. Two species of marine origin, *B. pinnipedialis* and *B. ceti*, were isolated from aquatic mammals [3]. *B. inopinata* [4] and *B. microti* [5] were gained from samples of a breast implant and common voles, respectively. *B. papionis* was described as eleventh species isolated from baboons (*Papio* spp.) [6], and recently, *B. vulpis* was isolated from the mandibular lymph nodes of red foxes (*Vulpes vulpes*) [7].

Brucellae are Gram-negative facultative intracellular stealthy pathogens. They can escape recognition of the innate immunity and avoid the intracellular destruction [8]. The virulence and pathogenesis of *Brucella* spp. are mainly associated with their survival and replication inside the host cells, including phagocytic and non-phagocytic cells. Brucellae are closely related, share noticeable similar genomes, but differ in their natural hosts, phenotypes as well as their immunogenic, proteomic and metabolomic properties [9,10]. To date, the mechanism behind host specificity is enigmatic. Host specificity might be caused by species-specific gene inactivation/activation that influences transcriptional regulators and outer membrane proteins [11]. *Brucella* lacks classical virulence factors and several aspects of its biology e.g., pathogenesis, survival in macrophages, and host specificity, are still not well understood. To explain its virulence and host specificity, a better understanding at the proteome and metabolome level could be helpful [12].

Currently, no safe human vaccine exists, while animal immunization is usually accomplished using live attenuated strains, such as *B. abortus* RB51 and S19, as well as *B. melitensis* Rev.1 [13]. Live attenuated vaccines have several drawbacks in animals such as residual virulence, abortion and interference with serology, and they are pathogenic for humans. In the last decade, various types of subunit vaccine candidates utilizing proteomic technology have been proposed. However, some problems regarding the levels of protection and safety remain a matter of open discussion [14].

The diagnosis of brucellosis remains challenging and is based mainly on the serology and isolation of *Brucella*. Serology basically relies on the detection of anti-*Brucella* lipopolysaccharide (LPS) antibodies. LPS-based assays have a low specificity due to cross-reactions resulting from the similarity between immunodominant epitopes of *Brucella* O-polysaccharide and those of other Gram-negative bacteria resulting in a false-positive reaction. Indeed, serology based on the use of a LPS antigen does not allow the discrimination of vaccinated and infected animals. This fact raised the need to replace the LPS antigen by protein antigens. The identification of genus- or species-specific protein candidates would be useful in designing diagnostics tools, which could diagnose *Brucella* infection and differentiate between infections caused by different *Brucella* species, respectively [15]. A comprehensive understanding of the intricate relation between the host cell and brucellae will improve the development of species-specific treatment and the design of better diagnostic tools and vaccines. The detection of prominent immunogenic proteins during the course of infection can help in the development of advanced diagnostic tools with high specificity and accuracy [16].

Proteomic technologies are major post-genomic approaches used to understand the consequences of regulatory processes on the protein composition of microbes. They have proven to be efficient tools to study the microbial physiology, gene expression and the interaction between the bacteria and the host cells [17,18]. The application of qualitative proteomics analysis has contributed to rapid species identification. A quantitative proteomic technique based on both gel and mass spectrometry approaches has been applied for a better understanding of the various aspects of the life cycle of the organisms. It has also aided in unraveling several enigmatic aspects of *Brucella* biology i.e., host-cell interaction, stress response, antibiotic targets and protein secretion [19], as well as the development of subunit vaccines and the identification of antigens for diagnosis. Here, we reviewed proteomic studies that have been carried out in brucellae with a focus on rapid species identification and improvement of serodiagnostics.

## 2. Rapid Species Identification of *Brucella* Using Matrix-Assisted Laser Desorption Ionization—Time of Flight Mass Spectrometry (MALDI—TOF MS)

The regular diagnosis of brucellosis relies on the indirect detection of anti-*Brucella* antibodies in milk by the milk ring test (MRT) as a field test, or by serological tools such as the slow agglutination test (SAT) and the rose bengal test (RBT), which are confirmed by the complement fixation test (CFT) and the enzyme-linked immunosorbent assay (ELISA) [20]. One of the main diagnostic challenges in serology is the cross-reaction resulting from the similarity of the O-antigenic side chain of LPS of *Brucella* to other Gram-negative bacteria. Thus, the diagnosis of *Brucella* based on sero-reactors should be handled with extreme caution unless accompanied by bacteriological and molecular diagnosis. Isolation, the classical biotyping and identification of *Brucella* spp., remains the gold standard for diagnosis, but it is hazardous, laborious, time-consuming and requires well trained personnel [21]. The genus-level identification relies on the classical bacteriological analysis of cultured colonies and DNA-based molecular tools [22]. The characterization of *Brucella* at the species and biovars level requires complete bacteriological and biochemical testing or advanced molecular techniques e.g., AMOS-PCR (an *abortus*, *melitensis*, *ovis* and *suis*-PCR) [23], multiplex PCR assay (Bruce-ladder) [24], multiple-locus variable number tandem repeat analysis (MLVA) [25], and whole genome sequencing (WGS) technology [26]. Both bacteriology and molecular assays require expertise, are time-consuming and laborious. Besides, the resolution of band sizes restricts existing PCR assays used for the detection of *Brucella* species on a gel [27].

In the past two decades, matrix-assisted laser desorption ionization—time of flight mass spectrometry (MALDI—TOF MS)-based typing, intact protein profiling (IPP) or intact-cell mass spectrometry (ICMS) have emerged as rapid species identification methods in the routine diagnostic laboratories [28,29,30,31,32,33,34,35]. In this method, a crude microbial sample is directly transferred on to the MALDI target plate, overlaid with UV-absorbing matrix solution, dried, the spectra is measured in a broad *m*/*z* range (2000–20,000 kDa) and the species identification is performed by pattern-matching with the database of reference spectra of known microorganisms [36]. The accuracy of the method is dependent on the availability of high-quality reference spectra, which is usually created using the most reproducible peaks (usually 70 peaks in the range of 2000–20,000 *m*/*z*) of high abundant proteins and ribosomal proteins specific to the species [37]. As mentioned, there is a minimal or no sample preparation step involved as culture colonies are directly transferred, proven rapidness (2–5 min from culture plate to species identification), the comparable accuracy to that of molecular methods and the high-throughput analysis and high-level occupational safety. Brucellae are identified by comparing the obtained MS spectra from each isolate to the reference spectra from a reference library [32]. For *Brucella*, taxon-specific mass spectral databases and peak pattern matching software tools are available now but additional reference spectra data on biovars are required to be created. Among several commercial software tools, the database integrated into the VITEK MS of the BioMerieuxbecame the first *Brucella* database validated for diagnostics with accreditation and is accessible to all users in routine diagnosis in 2010. A MALDI Biotyper (Bruker) was used to create reference spectra databases for *B. melitensis, B. abortus, B. suis, B. canis, B. ceti* and *B. pinnipedialis* [34]. The accuracy and stability of the technique were re-evaluated by testing a comprehensive collection of field and reference strains of well known *Brucella* spp. including *B. abortus, B. melitensis, B. suis, B. canis, B. ceti, B. inopinata, B. microti, B. ovis, B. pinnipedialis* and *B. neotomae* [31]. At the species level, 99.3% of 152 and 92% of 104 isolates were correctly identified. Misidentifications for *B. abortus, B. melitensis, B. suis* and *B. ceti* were reported to be very low, while incorrect biovar assignments were found frequently except for *B. suis* [31,32]. Constructing a reference library based on genetic relationships according to MLVA data improved the accurate identification of *Brucella* species with MALDI—TOF MS [32].

The difference in peak intensities due to growth conditions of the bacteria or the values of protein concentration was reported to be the least influential on species identification as the MALDI—TOF MS software tools considered only the most reproducible peaks of the generated spectra. Further improvements in terms of protein extraction protocols and the creation of unique brucellae reference databases have been reported [38]. In a recent study, a MALDI—TOF MS reference database was created and reported to identify the main classical human pathogens *B. melitensis*, *B. abortus* and *B. suis* at the species level with an accuracy of 100%, 92.9% and 100%, respectively [39].

As shown in Table 1, several reference databases have been created including a significant amount of type and field strains to enhance the accuracy of identification. The techniques and procedures are cost-effective after the initial investment on the instrumentation and the software. Hence, the reliability of the identification is based on the content and quality of the library, as well as other factors such as the protocol used for the preparation of the samples and the extraction of the proteins, the purity of colonies and culture conditions. MALDI—TOF MS was successfully applied to identify brucellae at the species level with cutoff values as per the recommendations of the software providers. However, it is still unable to identify the various biovars. The greatest drawback of MALDI—TOF MS was the requirement of pure colonies, which requires cultivation. Consequently, it is not a tool suitable to detect only a few bacteria usually present in clinical samples such as cerebrospinal fluids, serum or contaminated culture.

Recently, a liquid chromatography-tandem mass spectrometry (LC—MS/MS)-based method has been developed as a safe, highly accurate and unprejudiced identification method to identify highly pathogenic bacteria including *Brucella* [40]. This tool is based on analyzing the proteome and enables the rapid identification of *B. abortus*, *B. melitensis* and *B. suis* directly from positive blood culture flasks. The developed LC—MS/MS-based method makes use of discriminatory peptides. It considerably reduced the time required to identify the causative agents of bacteremia, and can quickly confirm the diagnoses of pathogens with a high level of accuracy.

## 3. Gel-Based Quantitative *Brucella* Proteomics Analysis

**Figure 1:**
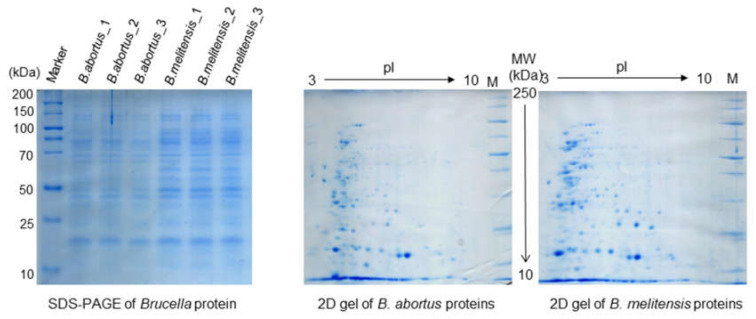
SDS-PAGE and two-dimensional gel electrophoresis (2DE)-stained gel with Coomassie prepared at our laboratory, each well containing 10 µg of proteins of *B. abortus* and *B. melitensis*, while each 2DE gel contains 150 µg of acetone-precipitated cell lysate of *B. abortus* and *B. melitensis* and showing the levels of various individual proteins.

Proteomics analysis using sodium dodecyl sulfate-polyacrylamide gel electrophoresis (SDS-PAGE), two-dimensional gel electrophoresis (2DE), difference gel electrophoresis (DIGE) and MALDI—TOF MS remains the gold standard for protein separation and the quantification of proteins from various samples. At the end of the 20th century, SDS-PAGE profiles (protein separation based on molecular weight) and immunoblotting were used in early studies of *Brucella* taxonomy [47], and to describe various proteomes of different *Brucella* species [48,49,50,51,52,53,54]. The initial idea was to isolate and characterize the outer membrane proteins of *B. abortus* and to compare those proteins with those of other vaccines and virulent strains [48,49]. Comparative analyses of outer membrane proteins extracted from the field strains of *B. ovis* and *B. melitensis* were investigated to identify an effective subcellular vaccine for ovine brucellosis [51]. Even though the SDS-PAGE proved that each species has a specific protein band pattern, the identification of a single protein was nearly impossible as each band was composed of several proteins of the same molecular weight (Figure 1). The technological improvement led to two-dimensional electrophoresis—Western blotting (2DE—WB) and mass spectrometry for protein identification. Two-dimensional electrophoresis—Western blotting has the ability to separate and display thousands of single proteins [55]. This technique provided much information about the proteins of complex samples. In the 2DE, proteins are separated based on their net charge and their molecular weight, and proteins are resolved as spots spread down in the gel. Those spots are representing various individual proteins (Figure 1). The combination of one- and two-dimensional gel electrophoresis and cellular immunoblotting were applied to improve protein identification and to compare the similarities of protein profiles among different *Brucella* spp. [52]. Two-dimensional electrophoresis—Western blotting, in combination with protein microsequencing, was used to map *B. melitensis* proteins. Comparative studies of protein expression for other brucellae followed [56,57]. Subsequently, this technology was used to study the pathogenesis of *Brucella* and to investigate its intracellular survival and its resistance strategy within macrophages [58]. To identify virulence-associated proteins in *B. abortus*, whole proteomes of virulent and of S19 vaccine strains were compared [59]. At the beginning of the 21st century, peptide mass fingerprinting, which employs MALDI—TOF MS, was applied to identify proteins that resulted in the rapid and large-scale mapping of whole brucellae proteomes. The mapping of complete *Brucella* proteomes, comparative proteomic analysis between different *Brucella* spp. (virulent and vaccine strains), and the examination of the differences in the protein profiles of *Brucella* grown under different conditions was investigated in a broad spectrum and on a large scale [45,46,47].

**Figure 2:**
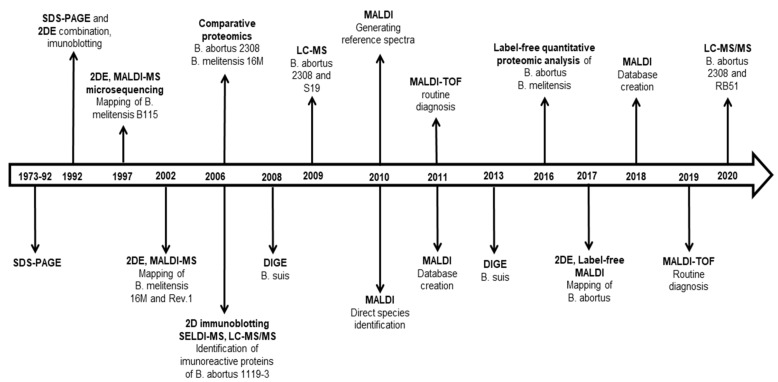
Chronological application of proteomics technology in *Brucella* research. SDS-PAGE (sodium dodecyl sulfate-polyacrylamide gel electrophoresis), 2DE (two-dimensional gel electrophoresis), DIGE (difference gel electrophoresis), SELDI-MS (Surface-enhanced laser desorption/ionization- mass spectrometry), MALDI-MS (matrix-assisted laser desorption/ionization—mass spectrometry) and LC-MS (Liquid chromatography–mass spectrometry).

To date, our understanding of the *Brucella* proteome is limited. Differences at the proteomic level of brucellae have been investigated with different growth conditions like temperature or acidity, and oxidative or nutritional stress [60]. In 1997, the mapping of *B. melitensis* B115 proteins was done using 2DE. The 2DE proteins map revealed 595 silver-stained protein spots and was considered the basis for comparative studies of protein expression in *Brucella* at that time [56]. The fast technical progress in the use of proteomics started in the early 2000s (Figure 2). For instance, a comparative proteomic analysis of a *B. melitensis* strain 16 M was done to identify and characterize the proteins expressed under laboratory conditions. Two-dimensional gel electrophoresis was used for protein separation. Of the 883 observed protein spots, 440 were identified by MALDI—TOF MS in a laboratory-grown culture. The consideration of the proteomic data with genomic sequences should lead to the identification of biochemical pathways associated with host specificity, pathogenicity, stress responses and virulence. The protein list of that study became the 2DE protein map to serve as a reference for subsequent *Brucella* proteomic studies [61]. Subsequently, the protein mapping of *B. melitensis* 16M was done to highlight the differences in protein expression with the attenuated *B. melitensis* Rev.1 vaccine strain. Certain metabolic pathways as well as the up and down-regulation of protein expression specific to each strain were studied, e.g., the differences in the expression of immunogenic 31-kDa outer membrane protein in Rev. 1 strain. This protein plays a role in sugar and amino acid binding, iron acquisition and lipid degradation [62,63]. Quantitative and qualitative differences in the protein expression patterns between two well-known human pathogens, *B. melitensis* and *B. abortus*, were studied by 2DE and peptide mass fingerprinting via MALDI—TOF MS using laboratory-grown reference strains *B. melitensis* 16M and *B. abortus* 2308 [64]. To understand the mechanism of *B. abortus* infection, the proteins secreted into the cultural supernatant were studied under laboratory conditions. More than 27 soluble immunogenic proteins were detected by 2DE and mass spectrometry. Those proteins induced strong humoral and cell-mediated immune responses and could promote antibody production leading to enhanced host defense against subsequent bacterial infection [65]. DIGE allowed the quantitative characterization of the intramacrophagic proteome and had sufficient discriminatory power to identify *B. suis* and host cell proteins connected to infection [66]. This approach helped to quantify the intracellular proteomes of different pathogens in a specific host cell. Around 168 proteins were altered in comparison to the extracellularly grown bacteria, and 44 proteins involved in bacterial metabolism were significantly regulated at the late stage of infection. A comparative proteome analysis of *B. abortus* 2308 and its virB type IV secretion system mutant using 2DE and mass spectrometry helped to identify new T4SS-related candidate proteins [67]. To investigate the regulative processes of *B. suis* survival under extreme nutrient starvation, DIGE provided a quantitative analysis of proteomes after long-term nutrient starvation of *B. suis* [68]. Thirty proteins were regulated in comparison to the bacteria grown in a rich medium and 70% of proteins involved in the adaptation to harsh conditions, transport and regulation showed over expression.

Proteomics analysis using 2DE and MALDI—TOF MS revealed that the changes in protein abundance of the infected host cells appeared between 48 and 96 h post-infection [69]. In recent years, attempts were made to better understand host-microbe interactions. The 2DE and mass spectrometry were used to identify proteins in the lung tissue of BALB/c mice challenged by aerosolized *B. melitensis* bacteria. Twelve differentially expressed proteins were involved in the infectious process of lung tissue [70]. In contrast to the SDS-PAGE, in the 2DE, the proteins could be separated, and the spots could be quantified and analyzed by MS, but the technique has a limited dynamic range. It is difficult to separate the low abundant proteins or very large proteins, and the technique requires a large amount of sample. Moreover, due to the staining sensitivity, some protein spots are difficult to visualize, and only Coomassie staining can be easily removed easily from the gel for further identification by MS. Thus, the attempts to use gel-free tools followed.

## 4. Mass Spectrometry Based Quantitative *Brucella* Proteomics

Proteomic analyses of various strains of *Brucella* using gel-based tools have been reported, suffering from limitations in terms of technology and the low number of identified proteins. Recently, a comparative proteomic analysis was carried out using LC—MS based on peptide fingerprinting [15]. A significant amount of data is now available to study the differences between the strains that are associated with virulence. Multidimensional scaling of differentially expressed peptides of *B. abortus* 2308 and S19 during intracellular infection was analyzed by LC—MS. The results provided insights into mechanisms utilized by brucellae to establish intracellular infection [71]. The label-free quantitative proteomic analysis of *B. abortus* and *B. melitensis* outbreak strains from a cow and a sheep, respectively, revealed 402 differentially expressed proteins. Among them, 63 and 103 proteins were detected exclusively in the whole-cell extracts of *B. abortus* and *B. melitensis*, respectively [15]. The mass spectrometry-based label-free relative quantitative proteomics analysis of *B. abortus* was reported to have identified a total of 1221 differentially expressed proteins following multiple environmental stresses and adaptations [72]. Furthermore, the proteogenomic mapping of *B. abortus* 104 M human vaccine strains using high-resolution mass spectrometry revealed 1729 proteins and 218 hypothetical proteins [73]. The liquid chromatography/tandem mass spectrometry was used to analyze secretory proteins from the *Brucella* rough mutants ΔrfbE and ΔrfbEΔvirB123. In this study, 861 unique proteins were identified, among which 37 were differential secretory proteins [74]. The proteomic analysis of membrane blebs of *B. abortus* 2308 and RB51 using liquid chromatography—tandem mass spectrometry (LC—MS/MS) succeeded to identify 220 and 171 proteins, respectively [75]. LC—MS requires skilled personnel to set the system, and it is an expensive option, both in terms of the price of the unit and the running costs. However, if the instrument is present and after training, it is relatively easy to operate daily, it offers several advantages such as improved accuracy and precision, retrieves an important amount of information from samples in a short time and has a higher selectivity. Liquid chromatography coupled to a tandem mass spectrometry (LC—MS/MS) has recently become a more popular alternative for the quantitative pan-proteome analysis of *Brucella* [12].

## 5. Serological Proteome Analysis (SERPA)

Despite the high genomic similarity among *Brucella* species [76], it has been demonstrated that they evoke different immune responses in natural hosts and display different protein expression profiles [15,77,78]. The identification of immunodominant proteins from brucellae was the target of several studies in recent years (Table 2). Looking for such proteins is required to increase the specificity of serodiagnostics and the development of safe subunit vaccines. Most of the studies on *Brucella* immunoproteomics have focused on the use of reference strains and experimentally produced hyper-immune sera [79,80,81,82,83]. However, using fully virulent field strains in proteomic studies is rarely done [15,78]. As shown in Table 2, using different protein lysates and different anti-*Brucella* sera revealed different spectra of immunoreactive proteins. When a protein sample of *B. melitensis* B115 was tested using monoclonal antibodies (MAbs), 25 protein spots out of 595 spots separated on a 2DE plot were stained [56]. The protein spots were identified as 89 kDa outer membrane protein, bacterioferritin, DnaK, CP24 and BP26. The same group tested protein preparations from *B. ovis* with a serum of a sheep naturally infected with *B. ovis.* The combination of 2DE and protein microsequencing succeeded to identify 82 immunoreactive cytoplasmic, periplasmic and membrane protein spots [57]. When the *B. abortus* 1119-3 whole-cell lysate was tested with rabbit hyperimmune sera, 17 out of 383 protein spots were stained. Using the surface-enhanced laser desorption/ionization mass spectrometry (SELDI-MS), these immunogenic spots were assigned to six proteins: Cu-Zn SOD, BCSP31, GroEL, GroES, L7/L12 and DnaK [79]. To identify novel candidate proteins for the development of a safer and efficient vaccine, immunoproteomics of the *B. abortus* 2308 cell envelope (CE) using antiserum from bovine and a human patient infected with *B. suis* was carried out. Among the 163 identified proteins, several new immunogenic proteins such as fumarate reductase flavoprotein subunit, cysteine synthase A and F0F1-type ATP synthase α-subunit were found. The authors assumed that those proteins were suitable candidates for developing vaccines against brucellosis in humans and bovines [80]. Thirty-two immunogenic spots reacted with both human and bovine anti-*Brucella* sera. A lysate of the *B. melitensis* M5 vaccine strain was tested against pooled bovine sera, and 88 immunoreactive protein spots were detected and assigned to 61 proteins by MALDI—TOF MS, including elongation factor G, F0F1 ATP synthase ß-subunit beta and OMP1. Only eight were virulence-related proteins [83]. A *B. melitensis* 16M cell lysate tested against pooled human and goat antiserum produced 23, 33 and 11 immunoreactive protein spots with sera of humans, goats or both, respectively. Two interesting riboflavin synthase α chain (RS-α) and Loraine synthase (LS-2) proteins were detected using antisera obtained from *Brucella*-infected humans and goats. Both proteins (rRS-α and rLS-2-) provided partial protection in mice challenged with *B. melitensis* [82]. Sera from cattle experimentally infected with *B. abortus* 2308 revealed 18 immunogenic insoluble proteins. The spots were reactive against anti-*Brucella* antisera but not towards negative sera and anti *Y. enterocolitica* sera [81]. It is worth mentioning that protein expression profiles differ depending on host species, *Brucella* spp. and stage of infection. Routine serodiagnosis fails to detect the stage of infection, but proteomic analysis shows time-course-dependent *B. abortus* protein patterns when a subsequent set of sera is used [84].

Prominent immunogenic proteins show time course-dependent reactions. It was found that 13, 24 and 55 proteins of *B. abortus* 544 were found to be reactive at day 10, 30 and 60 post-infection when tested with sera from experimentally infected mice in 2DE immunoblotting, respectively [84]. In the same context, *B. abortus* 544 protein lysates have been tested with sera from *B. abortus*-infected cattle at week 3, week 7 and week 10 post-infection. Thus, 134, 110 and 106 proteins were reactive, respectively, and 55 antigens were predominant at all three-time points [16]. Therefore, including different *Brucella* biovars and different sets of sera is essential to learn about the brucellae proteome and host immune responses to promote the development of advanced diagnostics and novel vaccine candidates [16,84].

Immunoblotting of whole-cell protein extracts of *B. abortus* and *B. melitensis* field strains against field sera collected from cattle, buffaloes, sheep and goats was performed with SDS-PAGE and Western blotting. The results showed that *Brucella* appeared to express heat shock proteins, enzymes, binding proteins and hypothetical proteins for their survival in the host during the early stage of infection [78]. When 2DE immunoblotting was used, 25 proteins of *B. abortus* and 20 proteins of *B. melitensis* were distinctly immunoreactive. Some were host-specific and others crossed the host species barrier. Three proteins from *B. abortus* (Dihydrodipicolinate synthase, glyceraldehyde-3-phosphate dehydrogenase, and lactate/malate dehydrogenase), and additionally, the amino acid ABC transporter substrate-binding protein from *B. melitensis* and fumarylacetoacetate hydrolase from both *Brucella* spp. were immunoreactive with the sera of naturally infected cattle, buffaloes, sheep and goats. The identified proteins were supposed to be used for the design of serological assays able to detect pan-*Brucella, B. abortus*- and *B. melitensis*-specific antibodies [15]. In addition to the number of immunoreactive spots detected in each study, there were few fainter signals on the Western blot membrane, which were not traceable on the Coomassie-stained 2DE gel. Some of the signals appeared fainter or even absent on the gel. This phenomenon might be caused by the sensitivity limitation of the staining/visualization method. The proteins behind these weak signals might explain the mechanism of host specificity and the molecular mechanism of intercellular survival.

Recently, several studies have been carried out to evaluate immunogenicity and protective responses, as well as the application of immunogenic proteins in the diagnosis of brucellosis [14,85]. For instance, the purified rAdk and rSecB of *B. abortus* have been proposed to be potential candidates for subunit vaccines [86]. However, it is important to consider that the protective responses observed in the mice model may not reflect the same protection in natural hosts. The immunogenic properties of BP26 and BLS proteins have been tested and proposed to be among the best candidates for serology [87]. However, some studies showed optimistic results, the potential use of protein antigens in diagnosis and subunit vaccines under field conditions is very limited.

## 6. Summary and Perspective

Proteomic technologies are major post-genomic approaches used to understand the consequences of regulatory processes on the protein composition of microbes. It has proven as a handy tool to study the microbial physiology, gene expression and the interaction between bacteria and their host cells. Since the beginning of the 21st century, proteomics technology has been applied to unravel various enigmatic areas of *Brucella* research. The earliest works begun with the protein separation by SDS-PAGE and 2DE, i.e., the manual and mechanical excision of protein spots and the identification of proteins using MALDI—TOF MS. In recent years, LC- MS-based proteomics technology has been developed and is widely applied in *Brucella* research. It is capable of identifying and quantifying higher numbers of proteins. Immunoproteomics utilizing 2DE—Western blotting is one of the most common tools implemented to identify antigens for serodiagnostics and the development of recombinant vaccine candidates. In the last decade, the development of LPS-free and protein-based antigens for serodiagnostics or vaccine design has attracted researchers worldwide and became a central target in the *Brucella* research. Several cell surface proteins and intracellular components of different *Brucella* spp. have been identified and classified as protective antigens. However, they most often provide only weak immunogenic responses. Several databases have been developed to be used for the direct identification and classification of brucellae. Now the technology is used in routine microbiological work and is the first line diagnosis. The use of proteomics as a comprehensive biological approach has contributed to rapid species identification and a better understanding of various aspects of the life cycle of brucellae. However, the current knowledge on proteomes of *Brucella* is mostly based on the examination of type strains or a limited number of isolates from a single host. This lack of data results in a somewhat sketchy picture of the whole proteome of *Brucella* and needs to be closed by pan-proteomics studies. Thus, the future of *Brucella* proteomics should focus on whole and pan proteomes [12]. Comparative and comprehensive proteomics analysis of several types and wild strains of brucellae from different host populations may help to discover crucial virulence proteins, unravel the secrets behind host specificity and the host–pathogen interaction phenomena, as well as to identify virulence mechanisms. This will also help to develop strategies to hinder spillover. Several aspects of the biology of *Brucella* like host specificity, the interaction with the host immune system, the development of resistance or intracellular survival are enigmatic. The use of proteomic approaches has already contributed to unravel and elucidate some aspects of the pathogenesis of *Brucella*. Proteome studies involving isolates of virulent and avirulent *Brucella* species and biovars in combination with multitudes of infection models will enable us to develop better diagnostics and safer vaccines in the future to combat brucellosis in humans and animals successfully.

## Figures and Tables

**Table 1:** List of studies utilizing matrix-assisted laser desorption ionization—time of flight mass spectrometry (MALDI—TOF MS) for the direct identification of brucellae.

No.	Year	*Brucella* spp. Used in Each Study	No. of Strains	Aim of the Study	Study
1	2020	*B. abortus, B. melitensis, B. suis*	3, 4 and 3	Diagnosis	[40]
2	2019	*B. abortus, B. melitensis*	29	Diagnosis	[41]
3	2019	*B. abortus*	5	Diagnosis	[42]
4	2018	*B. abortus, B. melitensis, B. suis, B. canis, B. canis, B. ovis, B. neotomae*	75	Database creation	[38]
5	2018	*B. abortus, B. melitensis, B. suis, B. canis*, *B. ceti, B. inopinata, B. microti, B. ovis, B. pinnipedialis,* and *B. neotomae, B. papionis*	84	Database creation	[39]
6	2017	*B. canis*	38	Diagnosis	[43]
7	2016	*B. melitensis* Rev-1 and *B. abortus* 19BA	2	Comparative analysis	[44]
8	2016	*B. abortus, B. melitensis, B. suis, B. canis, B. neotomae, B. inopinata, B. microti, B. ceti, B. ovis, B. pinnipedialis,* and *B. papionis*	15 reference strains	Protocol for sample preparation	[45]
9	2015	*B. canis*	1	Case diagnosis	[28]
10	2015	*B. melitensis* and *B. suis*	19	Diagnosis	[29]
11	2013	*B. abortus, B. melitensis, B. suis, B. canis,* B. *ceti, B. inopinata, B. microti, B. ovis, B. pinnipedialis,* and *B. neotomae*	104 field and 33 reference strains	Interlaboratory comparison	[31]
12	2013	*B. melitensis* and *B. suis*	6 and 3	Diagnosis	[46]
13	2012	*B. melitensis*	1	Sample preparation	[30]
14	2011	*B. abortus, B. melitensis, B. suis, B. canis,* B. *ceti, B. neotomae, B. ovis* and *B. pinnipedialis*	170	Diagnosis, Database creation	[32]
15	2011	*B. melitensis*	1	Case diagnosis	[33]
16	2010	*B. abortus, B. melitensis, B. suis, B. canis,* B. *ceti, B. pinnipedialis*	131	Generating reference spectra	[34]
17	2010	*B. melitensis*	1	Case diagnosis	[35]

**Table 2:** List of immunoproteomics studies on brucellae using different sets of anti-*Brucella* antisera showing the number of the immunoreactive proteins, the techniques applied and the year of publication.

No.	*Brucella* spp. Used	Source of Anti-Sera Used	No. of Immunoreactive Proteins Identified	Technique Applied	Year	Ref.
1	*B. abortusB. melitensis* clinical strains	Cattle, buffaloes, sheep and goats	25 proteins of *B. abortus* and 20 proteins of *B. melitensis*	2D immunoblotting, MALDI—TOF MS	2016	[15]
2	*B. abortusB. melitensis* clinical strains	Cattle, buffaloes, sheep and goats	Eight proteins of *B. abortus* and ten proteins of *B. melitensis*	SDS-PAGE Western blotting, MALDI—TOF MS	2015	[78]
3	*B. abortus* 544	*B. abortus*-infected cattle	134, 110 and 106 proteins were recognized at 3, 7 and 10 weeks of infection, and 55 common antigens	2D immunoblotting, MALDI—TOF MS	2015	[16]
4	*B. abortus* 544	Experimentally infected mice	13, 24 and 55 immunoreactive proteins were detected at 10, 30 and 60 days post-infection, respectively. MALDI identified 17 of the 67 proteins	2D immunoblotting, MALDI—TOF MS	2014	[84]
5	*B. abortus* 1119-3	Experimentally infected cattle with *B. abortus* 2308	18 immunogenic insoluble proteins showed immunoreactivity against positive sera	2D immunoblotting, MALDI—TOF MS	2012	[81]
6	*B. melitensis* 16 M and Rev.1	Pooled human and goat antisera	23, 33 and 11 proteins reacted with human, goat and both sera, respectively.	2D immunoblotting, LC—MS	2011	[82]
7	*B. melitensis* M5 vaccine strain	Pool of 15 bovine anti-*Brucella-*positive sera	88 immunoreactive protein spots assigned to 61 proteins. 12 are immunogenic, 8 are virulence-related proteins	2D immunoblotting, MALDI—TOF MS	2011	[83]
8	*B. abortus* 1119-3	Hyper-immune antisera from rabbits	17 immunoreactive out of 383 spots assigned to 6 proteins	2D immunoblotting, MALDI—TOF MS and SELDI-MS	2006	[79]
9	*B. abortus* 2308	Bovines and infected human patients with *B. suis* infection	42 and 23 immunogenic spots identified against human and bovine sera, respectively	2D immunoblotting, MALDI—TOF MS and LC—MS.	2006	[80]
10	*B. ovis*	Ram naturally infected with *B. ovis*	82 reactive protein spots were assigned to 21 proteins	2D immunoblotting and protein microsequencing	1997	[57]
11	*B. melitensis* B115	Monoclonal antibodies (MAbs)	25 protein spots from 595 protein spots separated by 2DE reacted	2D immunoblotting, N-terminal microsequencing	1997	[56]

Sodium dodecyl sulfate-polyacrylamide gel electrophoresis (SDS-PAGE); Surface-enhanced laser desorption/ionization mass spectrometry (SELDI-MS); Liquid chromatography—mass spectrometry (LC—MS).
